# Mental health rehabilitees’ agency construction and promotion in community-based transitional work programme

**DOI:** 10.1080/17482631.2023.2202972

**Published:** 2023-04-17

**Authors:** Miira Niska, Melisa Stevanovic, Henri Nevalainen, Elina Weiste, Camilla Lindholm

**Affiliations:** a Faculty of Social Sciences, University of Helsinki, Helsinki, Finland; b Faculty of Social Sciences, Tampere University, Tampere, Finland; c Finno-Ugrian and Scandinavian studies, University of Helsinki, Helsinki, Finland; d Finnish Institute of Occupational Health, Helsinki, Finland; e Faculty of Information Technology and Communication Sciences (Language Studies), Tampere University, Tampere, Finland

**Keywords:** Agency, Clubhouse, conversation analysis, discursive psychology, mental illness, transitional work

## Abstract

**Purpose:**

The integration of mental health rehabilitees into the labour market is an important policy objective everywhere in the world. The international Clubhouse organization is a third-sector actor that offers community-based psychosocial rehabilitation and supports and promotes rehabilitees’ state of acting and exerting power over their lives, including their (re)employment. In this article, we adopt the perspective of discursive psychology and ask how mental health rehabilitees’ agency is constructed and ideally also promoted in the Clubhouse-based Transitional Employment (TE) programme.

**Methods:**

The data consisted of 26 video-recorded TE meetings in which staff and rehabilitees of one Finnish Clubhouse discussed ways to further their contacts with potential employers. The analysis was informed by discursive psychology, which has been heavily influenced by conversation analysis.

**Results:**

The analysis demonstrated how rehabilitees adopt agentic positions in respect to TE-related future activities, and how Clubhouse staff promote and encourage but also discourage and invalidate these agentic positionings. The analysis demonstrated the multifaceted nature of agency and agency promotion in the TE programme.

**Conclusions:**

Although ideally, Clubhouse activities are based on equal opportunities, in everyday interaction practices, the staff exercise significant power over the question whose agency is promoted and validated in the TE programme.

## Introduction

High employment rate is a major societal goal in Europe. An important group of unemployed working-age people consists of those recovering from mental illnesses (OECD/EU, 2018). People with a mental illness are twice as likely to be unemployed as people with no mental illness (OECD, 2012). Work offers both personal and economic benefits and many mental health rehabilitees would like to join the labour force (Fekete et al., 2020, 2021; Goodwin & Kennedy, 2005; Kroezen, 2013; Pirttimaa & Saloviita, 2009). The problem is that the illness and the social stigma attached to it tend to impede their ability to find and retain employment (Cornwell et al., 2009; Krupa et al., 2009; Stuart, 2006). Societal pressure to find functional ways to promote mental health rehabilitees’ labour market integration is thus considerable (OECD/EU, 2018).

One important agent in mental health rehabilitees’ employment promotion is the international Clubhouse organization. The Clubhouse offers community-based psychosocial rehabilitation for people with mental illnesses. Their main concept involves the “work-ordered day” in which rehabilitees, also called Clubhouse members, participate in daily activities to maintain and develop the Clubhouse community, such as grocery shopping, staff recruitment and administration (Hänninen, 2016; McKay et al., 2018; Pirttimaa & Saloviita, 2009). In and through all this the Clubhouse organization promotes mental health rehabilitee’s *initiative* and *activity* (Fekete et al., 2020, 2021), *participation* (Tanaka et al., 2021) or *agency* (Valkeapää et al., 2019), that is, their state of acting and exerting power over their lives.

To promote rehabilitees’ agency over their own employment, the Clubhouse organization has a Transitional Employment (TE) programme. As part of this programme, the organization makes contracts with employers and trains their rehabilitee members for a part-time working period during which the rehabilitees can test their work ability and train work-related skills. The work period for an individual member is commonly between six and nine months. Clubhouse communities manage all TE workplaces, which means that individual Clubhouses oversee TE recruitment, training, and replacement processes. Clubhouse staff and rehabilitees gather regularly for TE meetings to generate and manage TE workplaces, which are crucial for the programme’s operation. (McKay et al., 2018; Pirttimaa & Saloviita, 2009; Valkeapää et al., 2019)

In this article, we ask how mental health rehabilitees’ agency is constructed in the context of the TE programme, and how Clubhouse staff members take part in the rehabilitees’ agency construction. We adopted the methodological approach of *discursive psychology* (Kent & Potter, 2014; Potter, 2013; Wiggins, 2017) and analysed the discussions between staff members and rehabilitees in Clubhouse-based TE meetings which took place in one Finnish Clubhouse. The aim of our study was to better understand the interaction processes in which mental health rehabilitees’ agency is constructed and ideally promoted in a community-based transitional work context. Before presenting the empirical study, we describe our methodological perspective in more detail.

## Discursive perspective on agency construction and promotion

Discursive psychology is a strand of discourse studies or discourse analysis that focuses on the interactional construction, management, and consequences of social psychological phenomena (Wiggins, 2017). As agency is among the key concepts of social psychology, it has been widely discussed in discursive psychology (e.g., Billig, 2009; Locke, 2008; Madill & Doherty, 1994; Niska & Vesala, 2013, 2015; Reynolds et al., 2007; Weatherall, 2020). In discursive psychology, agency, or the state of acting and exerting power, is something constructed in and through discourse (Potter, 2005).

According to discursive psychology, agency construction takes place in two ways. First, as people are active discourse users, agency can be understood as individuals’ state of acting in the sense of taking part in a conversation (Burr, 2003; Edley, 2001; Madill & Doherty, 1994). In the context of therapy, counselling and mental health rehabilitation, researchers have approached agency from the conversation analytic perspective, as actors’ ability to make proposals and resist others’ proposals (Ekberg & LeCouteur, 2020; Kushida & Yamakawa, 2020; Stevanovic et al., 2020). Conversation analysis is a stand-alone methodological approach to the sequential and detailed nature of talk and text, which has heavily influenced discursive psychology (Wiggins, 2017). Inasmuch as agency is understood as participation in a conversation, then *agency promotion* means action that encourages and strengthens other individuals’ participation in a conversation.

Stevanovic et al. (2020b, 2020, 2020a) have studied joint decision-making in the Clubhouse context and identified the practices by which the Clubhouse staff promote mental health rehabilitees’ agency in joint decision-making processes. First, the staff encourage rehabilitees’ responsiveness to the proposals they make. For example, the staff may remind the rehabilitees of their prior experience and knowledge, which enables them to comment on the proposal (Stevanovic et al., 2020b). The staff may also fine-tune the openness as opposed to the closedness of the form of their proposal, to provide the rehabilitees with genuine opportunities to contribute to the content of the decisions, while making participation easy enough for them (Stevanovic et al., 2020a). Second, in cases in which the rehabilitees make proposals, the staff may encourage further participation by enthusiastically elaborating on these proposals (Stevanovic et al., 2020). The staff may also occasionally retrospectively treat rehabilitees’ turns as proposals—even if the status of the turn as a proposal is not clear (Stevanovic et al., 2020b).

According to discursive psychology, agency construction also takes place in another way. When people use discourse (the first meaning of agency), they adopt and indicate agentic positions for themselves and other people (the second meaning of agency) (Niska & Vesala, 2013; Olakivi & Niska, 2017; Reynolds et al., 2007). A simple example of a turn in which agentic position is adopted is “I did it”. With this turn the speaker constructs personal responsibility over some occurrence by reporting the action as deliberate (Kurri & Wahlström, 2007). Davis and Harré (1990) talk about *reflexive positioning* and *interactive positioning*. In reflexive positioning, people use discourse to adopt positions for themselves (as in “I did it”). In interactive positioning, people use discourse to indicate positions for others (“you did it”). If agency is understood as an agentic position adopted in discourse, *agency promotion* means encouraging other individuals to use discourse specifically to adopt agentic positions with respect to some past, present or future action (e.g., Kurri & Wahlström, 2007).

Positioning processes have been the research topic in various therapy, counselling, and rehabilitation contexts (e.g., Etelämäki et al., 2021; Kurri & Wahlström, 2007; Seilonen & Wahlström, 2016; Toivonen, 2019), but to the best of our knowledge, no previous studies have focused on positioning processes in the community-based transitional work context. Researchers have been especially interested in “agentless talk” and demonstrated how therapy clients position themselves as not being in control of the described actions (e.g., Seilonen & Wahlström, 2016; Seilonen et al., 2012; Toivonen et al., 2019). In addition, previous studies have demonstrated how over time, therapy interaction gradually shifts clients’ positions from agentless to agentic ones (e.g., Avdi et al., 2015; Kurri & Wahlström, 2007).

Previous studies on positioning processes have mainly focused on agentic positioning in relation to past actions. The main interest has been whether patients or clients accept responsibility for past occurrences. In this study, our focus is on agentic positioning in relation to future action. Reflexive agentic positioning regarding some future action commonly takes place via I-formatted action proposals. These proposals may refer to one’s ability to act “I could make that call”, “I can visit that company”, or to one’s intentions, aims or purposes to act “I want to attend that meeting” (see Seilonen & Wahlström 2016; Weatherall, 2020). In this empirical study, we investigated (a) ways in which mental health rehabilitees adopt agentic positions towards future actions that further the aims of the TE programme and, thus, their opportunities of gaining employment, and (b) ways in which Clubhouse staff take part in, and ideally promote, rehabilitees’ agentic position construction. Before the results, we represent the data and methods.

## Materials and methods

The data we analysed in the study originated from a Finnish research project *Interaction, social inclusion, and mental illness*. In Finland, mental disorders are the most common condition leading to receipt of the disability pension (Finnish Centre for Pensions, 2020), and societal pressure to promote mental health rehabilitees’ (re)employment is understandably high. With 23 Clubhouses around the country, the Clubhouse organization is an important mental health rehabilitation agent in Finland (Finnish Clubhouses, 2021).

The data consisted of 26 video-recorded TE meetings, which took place weekly over an 11-month period, from October 2016 to August 2017 in one Finnish Clubhouse. In these meetings, the staff and rehabilitees discussed potential employers and advanced contacts between potential employers and the Clubhouse organization. The participants discussed future actions and distributed tasks such as sending information letters, calling potential employers and participating in employer meetings. The TE meetings involved one to six rehabilitees and one to three staff members. As the meetings were voluntary and open to all Clubhouse members the participants varied: some rehabilitees attended most meetings, others participated only once. Staff were trained in social work, and their work experience varied from approximately six months to several years. The duration of the meetings varied from 13 to 67 minutes (comprising a total of 794 minutes of interaction).

Research ethics approval was obtained from the Southern Finland Clubhouse Association and the board of directors at the relevant Clubhouse granted us research permits. The study was conducted in accordance with the Declaration of Helsinki. Written, informed consent was obtained from all participants. We have ensured the anonymity of the participants by altering all details that could help identify them in the main text and the data excerpts, which we present in the results section.

As discussed above, discursive psychology has been heavily influenced by conversation analysis, which uncovers interaction practices through detailed microanalysis of naturally occurring interaction situations (Edwards & Potter, 1992; Potter, 2010; Wiggins, 2017). The starting point for conversation analytic investigations is the observation that each utterance obtains its “meaning” in relation to the prior utterances (EA, 2007). Institutional conversation analysis builds on the basic ideas of conversation analysis but investigates how sequences of social actions contribute to achieving institutional goals (Arminen, 2005), such as promoting rehabilitees’ agentic positioning towards activities that further their opportunities to find employment.

We conducted the analysis using the original Finnish video-recordings and their transcripts (transcription symbols in the Appendix). In the first stage of the analysis, we watched the 26 video-recordings several times to identify all the sequences in which the rehabilitees’ agentic positioning towards TE-related future activities (e.g., “I can call”) was accomplished (e.g., Wiggins, 2017). This collection consisted of 31 sequences. In the second stage of the analysis, the sequences were analysed in detail. We investigated how agentic positions were constructed and paid special attention to the turns that preceded and followed the rehabilitees’ agentic positioning. The excerpts we present in the next section were translated from Finnish into English by the third author of this article. They were chosen because they illustrate particularly well the ways in which the agentic positions were constructed and promoted in the data.

## Results

### Rehabilitees’ reflexive agentic positioning: its encouragement and validation

The first two selected excerpts are examples of *reflexive agentic positioning* in which the rehabilitees adopt agentic positions for themselves (Davis & Harré, 1990). The agentic positions in relation to future TE-related activities were adopted by referring to their ability to act, for example, make calls to potential employers and take part in meetings and negotiations. The first reflexive agentic positioning took place in a TE meeting attended by several new participants. Excerpt 1 begins with a staff member’s (S1) act of acknowledging the presence of the new group members.

**Table:** Excerpt 1 (S1=staff, R1–R3= rehabilitees).

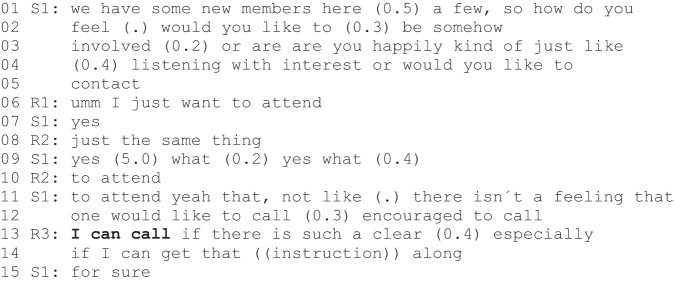

**Table:** Excerpt 2a (S1=staff, R1=rehabilitee).

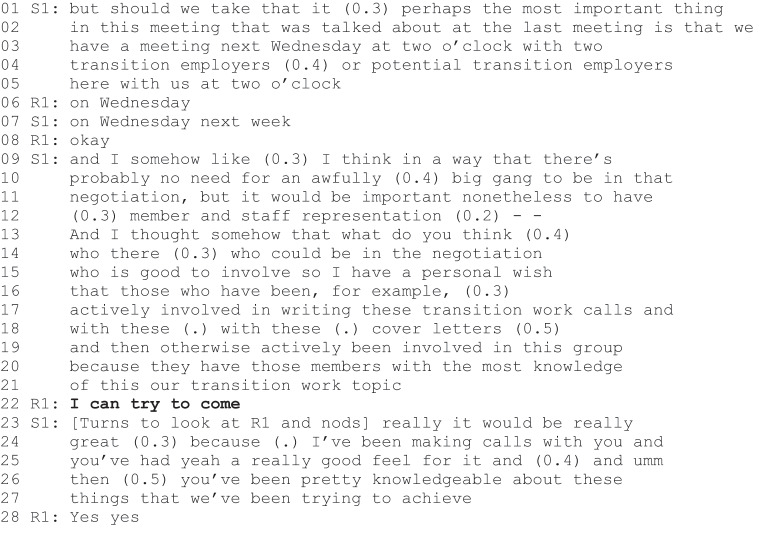

**Table:** Excerpt 2b (S1-S2=staff, R1–R3= rehabilitees).

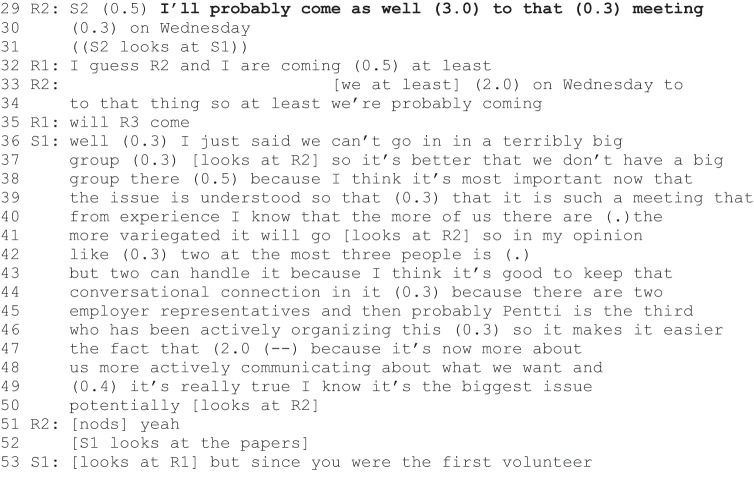

**Table:** Excerpt 3 (S1-S2=staff, R1= rehabilitee).

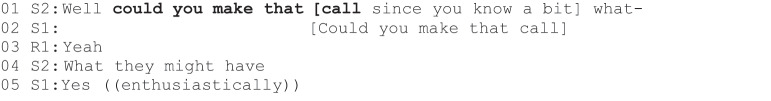

**Table:** Excerpt 4 (S1–S2=staff, R1=rehabilitee).

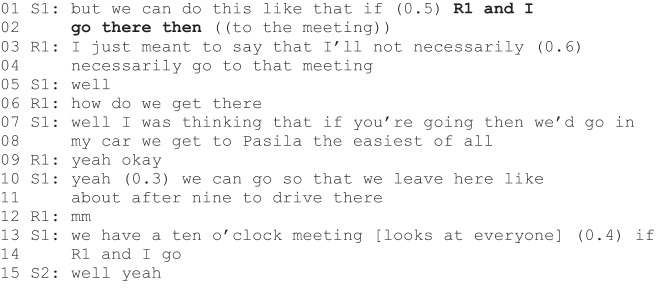

**Table:** Excerpt 5 (S1=staff, R1–R2=rehabilitees).

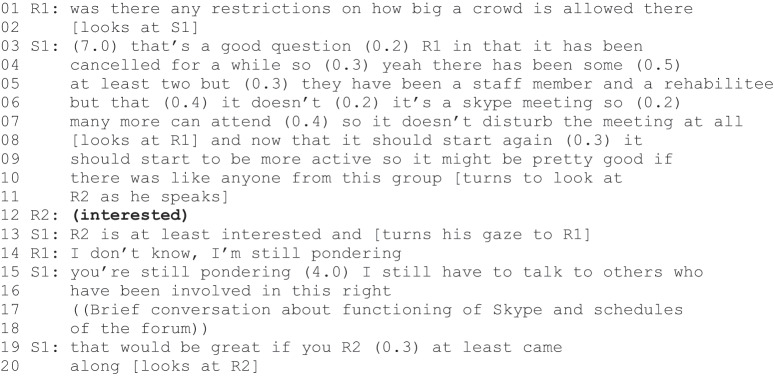

**Table:** Excerpt 6 (S1-S2=staff, R1–R3=rehabilitees).

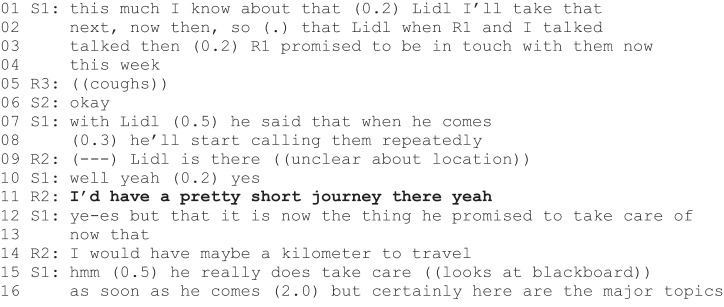

After acknowledging the presence of the new participants (line 1), S1 suggests two possible positions for them: an involved member or a passive observer. Although an “observer of the group” could be considered an agentic position, mere observation does not further the aims of the programme. After two new participants (R1 and R2) adopt the observer position (lines 6–10), S1 formulates a *zero-person construction* (Couper-Kuhlen & Etelämäki, 2015 also Etelämäki et al., 2021) “there isn´t a feeling that one would like to call” (lines 11–12). The turn could be interpreted as a reformulation of R1”s and R2”s turns, but reference to calling enables R3 interpretation of the turn as the proposition of an alternative “would like to call feeling”, which requires a response. Her response is consent formulated as an action proposal “I can call” (line 13). S1 enthusiastically validates the proposal with “for sure” (line 15).

The second reflexive agentic positioning takes place in a TE meeting in which the main topic to be discussed is an upcoming meeting with potential employers. R1 is late for the meeting but once he enters the room, S1, introduces the topic.

At the beginning of Excerpt 2a, S1 describes an upcoming meeting with two potential employers and proposes that at least one rehabilitee from the group would attend the meeting (lines 11–12). He continues his proposal, stating a personal wish that this/these rehabilitees would have been actively involved in contacting potential employers (lines 15–19). S1”s turn is followed by R1”s hesitant ‘I can try to come (line 22). S1 interprets the turn as an action proposal and indicates his satisfaction with a positive evaluation turn (“really it would be really great”, lines 23–24). He also shows his satisfaction by describing R1 as knowledgeable (“you’ve been pretty knowledgeable about these things that we’ve been trying to achieve”, lines 26–27).

An interesting difference between the Excerpt 1 and the Excerpt 2a is the way in which the members of staff engender the rehabilitees’ reflexive agentic positioning. In Excerpt 1, the staff member formulates a zero-person turn, which is not targeted at any single participant and allows all the rehabilitees, at least the new rehabilitees, to locate themselves as the zero-person (see also Ruusuvuori et al., 2020). In Excerpt 2a, the staff member formulates a proposal, which invites responses from only the active members of the TE group. Although S1 does not use a second person formulation, R1 is the only rehabilitee present who has been in contact with potential employers. It is reasonable to assume that S1 is aware of this. Two things further support the interpretation that in Excerpt 2, S1 is specifically promoting R1”s agency. First, positive evaluations, such as S1”s praise of R1 as knowledgeable, are common ways of supporting community involvement (Weiste et al., 2021). Second, the continuation of Excerpt 2 demonstrates how S1 explicitly invalidates another agency construction.

Instantly after R1’s action proposal and agentic positioning was validated (Excerpt 2a, lines 23–24), another rehabilitee, R2, declared his intention to act: “I’ll probably come as well to that meeting” (line 29). Although the turn includes hesitation (“probably”), it does not include modal verbs, which would indicate possibility (e.g., “I could”). In this sense, his participation appears self-evident, as if the speaker belongs to the group of active members that S1 constructed earlier (in Excerpt 2a, lines 15–19). Staff member S2, for whom R2 indicates his turn, looks at S1 and refrains from responding (line 31). In this situation, R1 validates R2”s proposal (line 32) and furthermore inquiries about R3”s participation (“will R3 come”, line 35). S1”s response is directed at R2 and the turn invalidates the original declaration. S1 announces that only two to three people should attend such a meeting (line 42). Nevertheless, S1 remains further accountable for this turn as it is not clear why he validated R1”s hesitant proposal to attend and invalidated R2’s declaration to attend. After a pause (line 52), S1 accounts for his action by referring to the order of the turns: R1 made the first proposal to attend the meeting (line 53).

In sum, the rehabilitees constructed agentic positions in relation to future action, referring to either their ability to act “I can make that call” or their intentions, aims or purposes to act “I’ll come”. The staff took part in the construction process in several ways. First, they made zero-person formulations which allowed numerous rehabilitees to take part in the conversation and adopt agentic (or non-agentic) positions in relation to the suggested activity. Second, they targeted their turns at a specific rehabilitee whose agency and involvement they wanted to promote and used positive personal evaluations. Furthermore, they discouraged and invalidated other rehabilitees’ agentic positionings.

### Staff’s interactive agentic positioning and action proposal interpretations

The third excerpt is an example of an *interactive agentic positioning* in which a staff member indicates the agentic position for a rehabilitee (Davis & Harré, 1990). This is done using a you-formatted proposal which refers to the rehabilitee’s ability to act, in this case, make a call to a potential employer. The first interactive agentic positioning takes place in a TE meeting in which the group has discussed a specific company and rehabilitee R1 has disclosed that he has worked for the company in the past.

After receiving information about R1’s past experience, S2 makes a you-formatted action proposal “well could you make that call” (line 1) which assigns an agentic position to R1. By acquiescing to S1’s proposal (“Yeah”, line 3), R1 accepts the positioning, which is enthusiastically verified by S1 (“Yes”, line 5).

In addition to you-formatted proposals, the staff members also assigned agentic positions to rehabilitees using we-formatted proposals. These proposals were both reflexive and interactive positionings in the sense that the staff members simultaneously adopted agentic positions for themselves and indicated agentic positions for the rehabilitees. The second interactive (and reflexive) agentic positioning takes place in a TE meeting in which the group has discussed an upcoming meeting with potential employers. This meeting is not the same as that discussed in Excerpts 2a and 2b.

Excerpt 4 begins with staff member S1’s proposal to attend the meeting with rehabilitee R1. S1 adopts an agentic position for himself but also indicates an agentic position for R1. Unlike R1 in Excerpt 3, R1 in Excerpt 4 does not acquiesce to the proposal but declares his ability to reject the proposal and the agentic position that follows (lines 3–4). However, instead of rejecting the proposal, he enquires about the mode of transport (line 6). After S1 promises they can drive to the meeting in his car, R1 acquiesces to the proposal (“yeah okay”, line 9). S1 requests validation from the other participants (“If R1 and I go” lines 13–14), and S2 validates his proposal (“Well yeah”, line 15).

In addition to interactive agentic positioning, the staff members take part in the rehabilitees’ agency construction by actively interpreting the rehabilitees’ turns as I-formatted action proposals (see Excerpt 1) – even when the status of the turn as an action proposal is unclear (see also Stevanovic et al., 2020b). Excerpt 5 is from a TE meeting in which S1 has introduced an upcoming seminar on transitional work.

At the beginning of Excerpt 5, rehabilitee R1 inquires how many people can attend the upcoming seminar. S1 answers R1”s question (line 5) but continues that the seminar will take place on Skype and thus anyone from the group could join in (lines 6–11). However, at this point, S1 turns their gaze to member R2 (line 10–11), who then makes a brief quiet comment, presumably saying he is interested (line 12). S1 treats R2”s turn as an action proposal and validates the proposal with a positive evaluation turn (“great if you R2 at least came along” lines 19–20).

Both interactive agentic positioning and action proposal interpretations are used in the data to support specific rehabilitees’ agency construction. Interestingly, the data also include a sequence in which a staff member first interprets a rehabilitee’s turn as an action proposal (as in Excerpt 5) and then invalidates the interpreted proposal. The next excerpt (Excerpt 6) is from a TE meeting in which the group has been discussing a list of potential employers who have not yet been contacted.

At the beginning of Excerpt 6, S1 talks about contacting a potential employer, Lidl, and makes an I-formatted action proposal (“I’ll take that next”, lines 1–2). However, he adds that he had discussed the Lidl with rehabilitee R1, who had promised to contact them (lines 3–4, 7–8). R2 then asks about the location of the shop and points out that he lives quite close (lines 9 and 11). S1 seems to interpret this turn as a proposal to contact the company because he invalidates R2”s turn, referring to R1”s proposal to be in contact (lines 12–13). S1”s interpretation is interesting, as R2”s turn could easily be interpreted as a hint that the shop would make a suitable TE option for him—not that he would like to contact Lidl. With the action proposal interpretation, S1 thus promotes R2”s agency as a discourse user (Stevanovic et al., 2020b) but impedes his agency as a future employer contactor. Much the same way in Excerpt 4, S1 invites everyone to validate his and R1”s agentic positions—and thus promotes their agency as discourse users but impedes their agency as future meeting participants.

In sum, the staff members took part in the rehabilitees’ agency construction with interactive positionings and action proposal interpretations. Agentic positions were assigned to the rehabilitees by you-formatted and we-formatted action proposals. Both turns assigned agentic positions to the target rehabilitees but did not necessarily promote their agency as discourse users and conversation participants. The rehabilitees naturally had the option of rejecting the staff members’ proposals, as demonstrated in Excerpt 4, but the action proposal was, in these cases, formulated by the staff member alone. In contrast, the staff members’ action proposal interpretations assigned agency to the rehabilitees over the proposal formulation and thus highlighted their agency as discourse users. Nevertheless, the staff also invalidated the interpreted action proposals, meaning that agentic positions in future activities were also discouraged.

## Discussion

In this article, we investigated agency construction in a community-based transitional work context. The TE programme of the Clubhouse organization is an example of third-sector organized mental health rehabilitees’ employment promotion. In the empirical study, we asked how mental health rehabilitees’ agency is constructed in TE meetings and how Clubhouse staff members take part in, and ideally promote, the rehabilitees’ agency construction process. We approached agency and agency promotion from the perspective of discursive psychology and conversation analysis. These theoretical-methodological approaches are highly useful in research that seeks to understand how institutional tasks are performed (or failed to perform) in institutional interaction. In the empirical study, we approached Clubhouse rehabilitees’ agency by focusing on their agentic positioning in respect to some future TE-related activity.

The data consisted of video-recorded TE meetings, in which staff members and rehabilitees from one Finnish Clubhouse discussed potential employers and ways in which to expand contact with these employers. Given the size of our data (26 video-recorded TE meetings, a total of 794 minutes of interaction), our collection of 31 sequences is not a huge one. In conversation analytic studies, the size of the collections typically ranges from a few dozen to hundreds of sequences. Thus, rehabilitees’ agentic positioning in respect to some future TE-related activity was not a frequent activity in the data. This is an interesting and somewhat surprising observation given that the TE programmes aim to promote rehabilitees’ agency over their own employment.

When the rehabilitees adopted agentic positions in respect to future activities, they used I-formatted action proposals and declarations (see Kurri & Wahlström, 2007). This reflexive agentic positioning took place when the rehabilitees offered to call potential employers or declared their willingness to participate in meetings. In the data, the staff used numerous ways to promote, encourage or engender the rehabilitees’ reflexive agentic positioning. First, the staff members encouraged the rehabilitees’ participation and positioning using zero-person formulated turns, which were open for numerous rehabilitees to respond to (also Ruusuvuori et al., 2020). Second, the staff members encouraged specific rehabilitees’ participation and positioning by giving verbal and non-verbal cues about whose involvement was desired. The target rehabilitees’ agency was also promoted by positive personal evaluations (also Weiste et al., 2021) and by discouraging and invalidating other rehabilitees’ agentic positionings.

In addition to encouraging the rehabilitees’ reflexive agentic positioning, the staff members took part in the rehabilitees’ agency construction by assigning them agentic positions using you-formatted and we-formatted action proposals. Interactive agentic positioning took place when staff members asked the rehabilitees to contact employers or proposed who should attend the meetings. Although interactive agentic positioning constructs rehabilitees’ agency in respect to specific future activity, it does not necessarily promote rehabilitees’ agency as active discourse users who take part in action proposal formulations. The final way in which the staff members took part in the rehabilitees’ agency construction was by interpreting their turns as action proposals—even when the status of the turn as a proposal was unclear (also Stevanovic et al., 2020b). In some cases, the staff members interpreted the rehabilitees’ inaudible, sporadic words as proposals for future action. Such interpretations naturally highlight the rehabilitees’ agency in proposal formulation. Nevertheless, the staff members also invalidated the agentic positionings that these interpretated proposals constructed. Although agency as a discourse user was highlighted, at the same time agentic positions in future activities were discouraged.

All in all, the analysis demonstrated the multifaceted and sometimes problematic nature of agency promotion in the TE programme. By promoting the rehabilitees’ agency in one sense (e.g., interactive positioning), the staff members ended up inhibiting their agency in another sense (e.g., active discourse user). By encouraging and validating one rehabilitee’s agency, the staff members simultaneously closed off agentic opportunities from the other participating rehabilitees (also Stevanovic et al., 2020). The analysis demonstrates that staff members exercise considerable power over the rehabilitees’ agency—both in terms of the type of agency and in terms of whose agency in the TE programme is promoted in the first place. This is important, considering that Clubhouse activities should be based on equality and equal opportunities (Fekete et al., 2020; Tanaka et al., 2021; Valkeapää et al., 2019).

Our aim is not to make moral judgements of the Clubhouse staff members’ actions. Conversation analytical research cannot be straightforwardly applied to instruct interaction (e.g., Stevanovic & Lindholm, 2016). Therefore, the purpose of our study is not to say how exactly Clubhouse staff members should or should not act in the TE meetings. However, conversation analytic research can enhance dialogue between research and professional interaction practices, and our study questions the simplistic assumption that TE programmes support rehabilitees’ agency in an equitable way.

Our empirical findings are interesting in relation to motivational interviewing (MI) interventions which have been found effective in improving vocational outcomes among people recovering from serious mental health illnesses (e.g., Hampson et al., 2015; Wewiorski et al., 2021). In line with the Clubhouse principles, MI seeks to support individuals’ own strengths and active participation by motivating them to make meaningful changes in their lives (Miller & Rollnick 2012; Hänninen, 2016). In MI, the professionals guide the conversation to strengthen clients’ change talk, for instance, by asking evocative questions and inviting reflection (Britt et al., 2022; Miller & Rollnick, 2012).

In our data, the staff members’ practices of interactive agentic positioning and encouraging the rehabilitees’ reflexive agentic positioning can be seen as micro-level aspects that contribute to rehabilitee’s agency construction and thus, possibly, promote positive change. However, the data also demonstrates the multifaceted nature of agency promotion. While promoting rehabilitees’ agency, the staff members also ended up inhibiting and suppressing rehabilitees’ agency. In line with prior MI studies (e.g., Britt et al., 2022), we highlight the importance of the practitioners’ interactional skills. Considering the fine-grained nature of the communicative practices, their reflective understanding is needed for supporting clients’ paths towards employment.

The study presented in this article naturally has some limitations. First, we analysed a relatively small dataset from one Finnish Clubhouse. For this reason, the analysis most likely did not capture *all* the ways in which agency could be constructed and promoted within the Clubhouse-based TE programme. Further research in other Clubhouses might uncover new ways of agency construction and promotion, but also new ways of agency suppression. Discursive psychology and conversation analysis, which approach language as social action, provide perfect tools for such studies. The data our analysis is based on represents a sample of interaction in one Clubhouse. This means that the results cannot be generalized to all Clubhouses in the world or even in Finland. The *potential* for described paradoxes in agency promotion exists, but such paradoxes might not realize in other Clubhouses’ interaction. Further research on TE programme interaction is needed to fully capture aspects of agency construction, promotion and suppression.

Second, the adopted methodological approaches of discursive psychology and conversation analysis focus on micro-level interaction. Although we can make claims about participants’ positioning in future actions, the study was unable to grasp the ways in which the mental health rehabilitees of the Clubhouse organization end up practising (or refraining from practising) their agency in the situations that were discussed in the data. Nonetheless, agentic positioning with respect to future activities can be considered a necessary but not self-sufficient predecessor of any agentic behaviour. On the way to (re)employment, mental health rehabilitees must be willing and able to adopt the position of an active work life agent, and our study demonstrates ways in which this position may or may not be achieved in the Clubhouse based TE meeting interaction.

## Appendix.

**Table:**

[]	Overlapping talk
(.)	A pause of less than 0.2 seconds
(0.0)	Pause: silence measured in seconds and tenths of a second
()	Transcriber could not hear what was said
((word))	Transcriber’s comments or descriptions
